# The Association Between Left Ventricular Hypertrophy and the Occurrence and Prognosis of Atrial Fibrillation: A Meta-Analysis

**DOI:** 10.3389/fcvm.2021.639993

**Published:** 2021-07-30

**Authors:** Huaqiang Xiang, Yangjing Xue, Zhi Chen, Yongwei Yu, Yangpei Peng, Jinsheng Wang, Kangting Ji, Huifen Zhu

**Affiliations:** ^1^Department of Cardiology, The Second Affiliated Hospital and Yuying Children's Hospital, Wenzhou Medical University, Wenzhou, China; ^2^Department of Medicine, Wenzhou Medical University, Wenzhou, China

**Keywords:** left ventricular hypertrophy, atrial fibrillation, predictor, prognosis, electrocardiogram

## Abstract

**Aims:** The aim of this study was to perform a meta-analysis of studies of the association of left ventricular hypertrophy (LVH) and atrial fibrillation (AF), especially the predictive and prognostic role of LVH.

**Methods and Results:** We searched Medline, Embase, and the Cochrane Library from inception through 10 April 2020. A total of 16 cohorts (133,091 individuals) were included. Compared with the normal subjects, patients with LVH were more susceptible to AF (RR = 1.46, 95% CI, 1.32–1.60). In patients with AF and LVH, there was a higher risk of all-cause mortality during 3.95 years (RR = 1.60, 95% CI, 1.42–1.79), and these patients were more likely to progress to persistent or paroxysmal AF (RR = 1.45, 95% CI, 1.20–1.76) than were patients without LVH. After catheter ablation of AF, patients with LVH were more likely to recur (RR = 1.58, 95% CI, 1.27–1.95).

**Conclusion:** LVH is strongly associated with AF and has a negative impact on outcome in patients with AF.

## Introduction

Atrial fibrillation (AF) is one of the major causes of stroke, heart failure, sudden death, and cardiovascular morbidity; it is the most common sustained arrhythmia worldwide. The number of patients with AF is predicted to rise steeply in the coming years (1). Hence, it is important to identify risk factors for AF as well as the outcomes associated with it. It has been demonstrated that left ventricular hypertrophy (LVH), detected by electrocardiogram or echocardiography, strongly predicted cardiovascular disease and its outcomes (2, 3). Likewise, Ramkumar et al. (4) stated that LVH detected by echocardiography would benefit the AF screening; Verdecchia et al. (5) indicated that LVH detected by electrocardiogram would improve risk stratification in anticoagulated patients with AF. To our knowledge, no study has systematically clarified the association between LVH and AF so far. Therefore, we performed a meta-analysis to comprehensively examine it. We sought to assess the predictive ability of left ventricular hypertrophy as risk factors for the development of AF, and we also attempted to determine whether the presence of LVH can identify that patients with AF have worse outcomes, including patients after the catheter ablation of AF.

## Methods and Results

### Search Strategy and Selection Criteria

**Figure 1:**
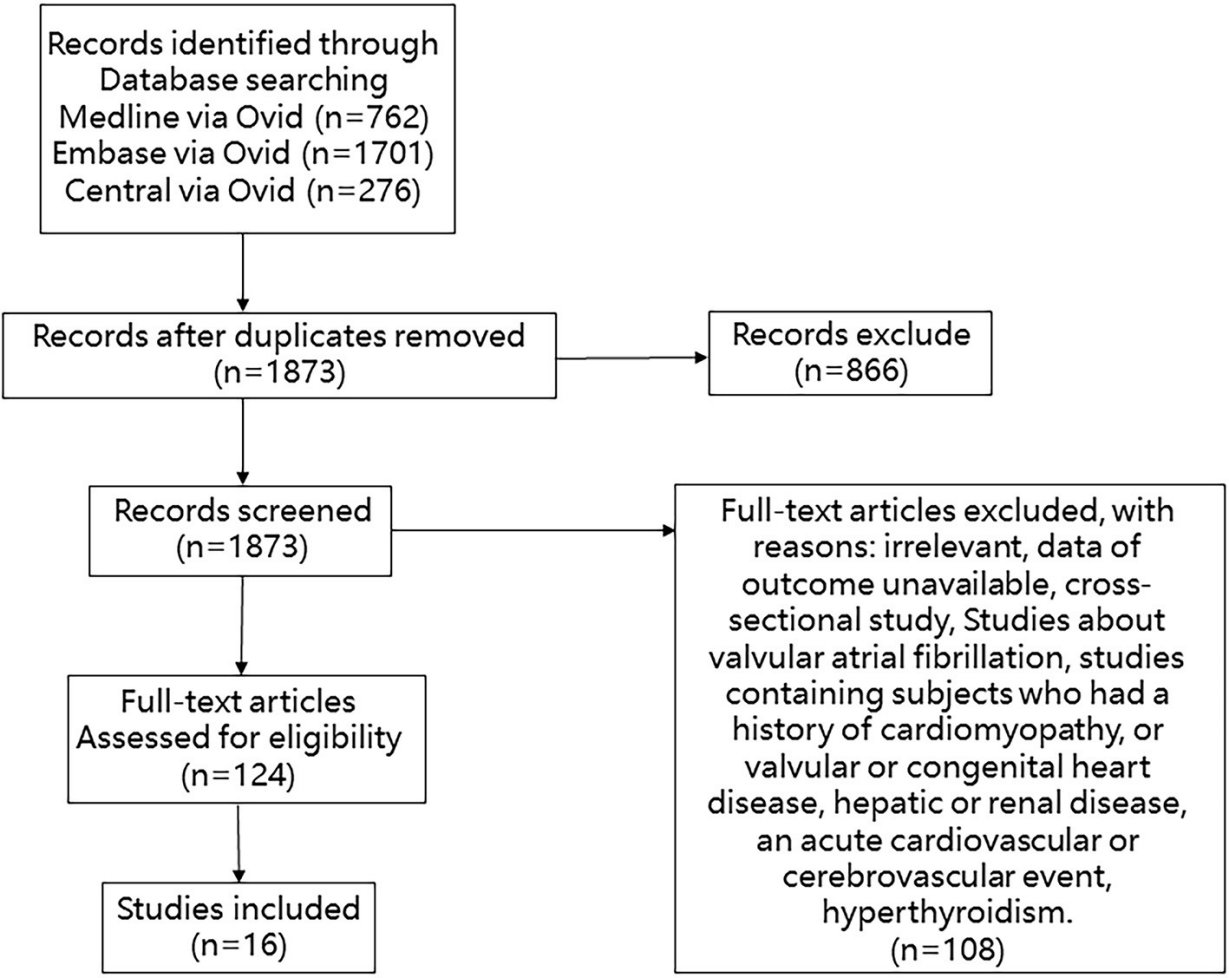
Flowchart of studies considered for inclusion.

We searched Medline, Embase, and Cochrane Library databases from inception through 10 April 2020 with the following terms: “atrial fibrillation,” “atrial flutter,” and “hypertrophy, left ventricular.” We manually searched for additional eligible studies in the reference lists of retrieved publications and relevant meta-analyses in the discipline. Studies were included if the patients they enrolled met the following criteria: (1) clear diagnosis of left ventricular hypertrophy assessed by echocardiography or electrocardiography at baseline; (2) data on total new-onset AF events or diseases related to prognosis including stroke, death, and cardiovascular events; and (3) data on the effect value such as the hazard ratio (HR) or relative risk (RR) between patients with or without LVH. Studies were excluded if their subjects had one of the following conditions: history of cardiomyopathy or valvular or congenital heart disease; hepatic or renal disease; acute cardiovascular or cerebrovascular event; and hyperthyroidism. Studies of valvular atrial fibrillation were excluded. Cross-sectional studies were excluded. The detail of the selection process is shown in Figure 1.

### Definition

Echocardiography-assessed LVH is preferably defined as LVMI ≥115 g m^−2^ in males and ≥95 g m^−2^ in females, but different cutoff points were also considered for eligibility. Electrocardiography-assessed LVH is based on a standard 7-lead or 12-lead ECG using a validated calculation [Sokolow–Lyon indices or Cornell voltage criteria (6)].

### Data Extraction and Quality Assessment

Two reviewers (Xiang Huaqiang and Xue Yangjing) independently screened titles and abstracts based on inclusion. After eliminating irrelevant studies, full text reports were reviewed. Case studies and review articles were excluded. Subsequently, we performed hand search of all included cohort studies until no further relevant studies were identified. Disagreements between the two reviewers were resolved by the third reviewer (Zhu Huifen). Then the two investigators (Xiang Huaqiang and Xue Yangjing) independently reviewed the main reports and supplementary materials and extracted information into an electronic database: study and patients characteristics, study design, interventions, the number of events of incident AF or diseases related to prognosis in each group, and the duration of follow-up. Any discrepancies regarding the extraction of data were resolved by an additional investigator (Zhu Huifen). The Newcastle-Ottawa Scale was used for the quality assessment of cohort studies. This scale appoints a maximum of nine stars to each study: four stars for the adequate selection of the two groups, two stars for comparability of groups on the basis of the design and analysis, and three stars for the adequate ascertainment of the exposure in both groups (7). Detailed data are presented in Table 1.

**Table 1:** Quality assuagement of cohort studies.

**References**	**Selection**	**Comparability**	**Exposure**
Jeong (8)	⋆	-	⋆⋆⋆
Hamada and Muto (9)	⋆⋆	⋆⋆	⋆⋆⋆
Renate et al. (10)	⋆⋆	⋆	⋆⋆
MacFarlane et al. (11)	⋆⋆	⋆⋆	⋆⋆
Chamberlain et al. (12)	⋆⋆⋆	⋆	⋆⋆⋆
Patel et al. (13)	⋆⋆⋆	⋆⋆	⋆⋆
Jonathan et al. (14)	⋆⋆⋆	⋆⋆	⋆⋆⋆
Lehtonen et al. (15)	⋆⋆⋆	⋆⋆	⋆⋆
Okin (16)	⋆⋆⋆	-	⋆⋆
Verdecchia et al. (17)	⋆⋆⋆	-	⋆⋆⋆
Padfield et al. (18)	⋆⋆⋆	⋆⋆	⋆⋆
Hijazi et al. (19)	⋆⋆⋆	⋆⋆	⋆⋆
Verdecchia et al. (5)	⋆⋆⋆⋆	⋆⋆	⋆⋆⋆
Badheka et al. (20)	⋆⋆⋆	⋆⋆	⋆⋆
De With et al. (21)	⋆	⋆	⋆⋆⋆
Im et al. (22)	⋆⋆	⋆	⋆⋆⋆
Akkaya et al. (23)	⋆⋆⋆	⋆⋆	⋆⋆⋆
Li et al. (24)	⋆⋆⋆⋆	⋆	⋆⋆⋆

### Data Analysis

Most of the studies we included presented adjusted HR in the published articles, and we self-calculated the RR from 2 × 2 tables for the remaining studies that did not. Effect estimates along with their 95% confidence intervals (CIs) by LVH were pooled results using fixed-effects models. The I^2^ statistic was calculated as a measure of the proportion of the overall variation that is attributable to the between-study heterogeneity. Analyses were conducted using STATA 14.

## Results

We included 16 studies in the end. At first, we included 18 cohort studies and 10 studies related to the predictive role of LVH in new-onset AF. In order to investigate whether left ventricular hypertrophy detected by electrocardiogram (ECG-LVH) would increase the risk of AF independently, we excluded one study that defined LVH via echocardiography. In addition, in six studies that investigated the role of LVH in the prognosis of AF, three studies were *post-hoc* analysis and two studies used the same original trials, thus, one of them was excluded. In short, nine studies investigated the predictive role of LVH, five studies investigated the prognostic role of LVH, and two studies investigated the difference of recurrence risk after catheter ablation of subjects with or without LVH.

### The Association Between LVH and New-Onset AF

**Figure 2:**
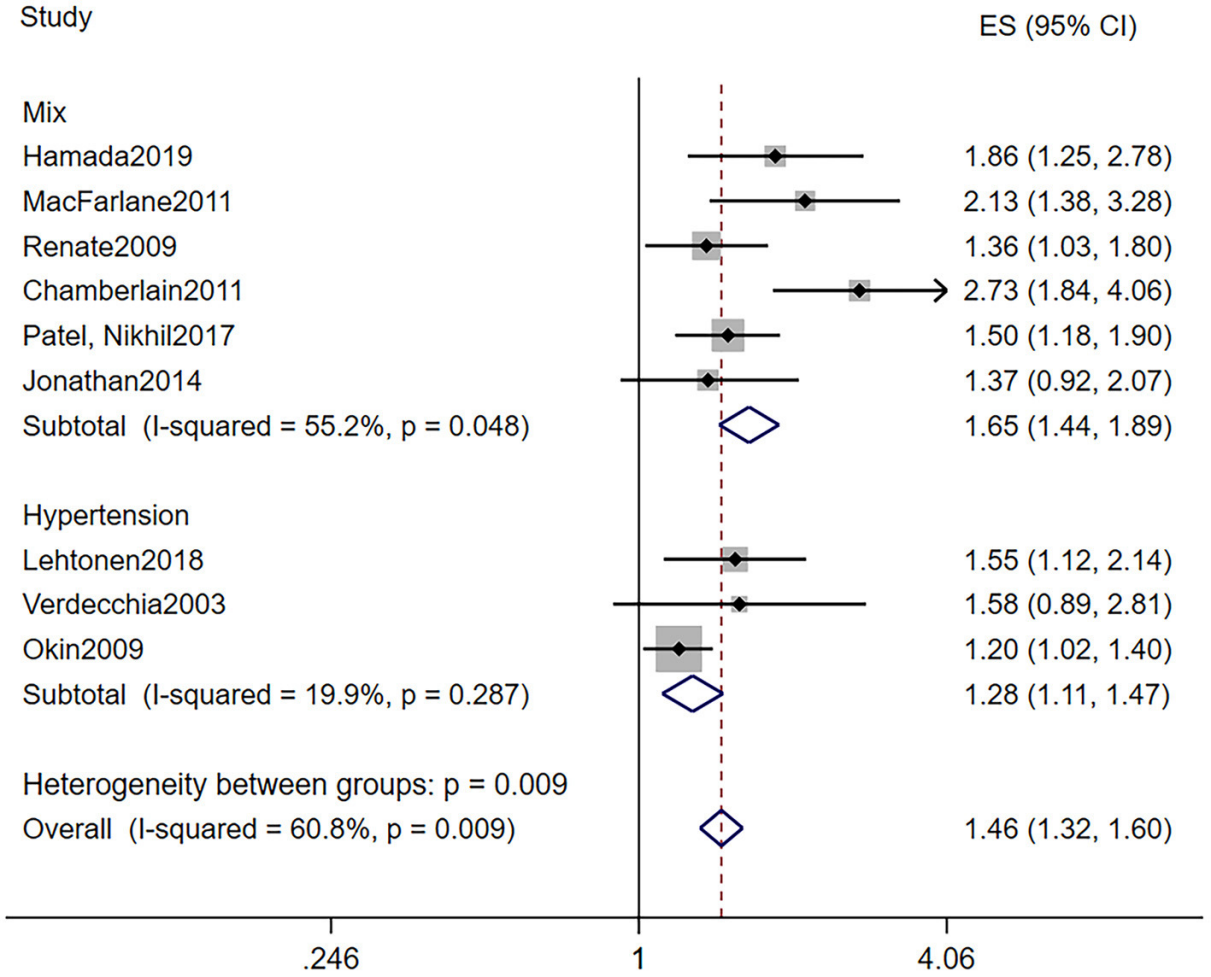
Different risk of new-onset AF in subjects with or without LVH. AF, atrial fibrillation; LVH, left ventricular hypertrophy.

The baseline characteristics of patients who took part in the research investigating the predictive role of LVH are shown in Table 2A. LVH was all defined by electrocardiogram. Particularly, we can divide the nine studies into two subgroups according to baseline blood pressure. Six out of the nine studies were based on the population including both hypertension and normotension, while the remaining three studies were based on hypertension patients only. In all, the pooled population consisted of 118,195 subjects, in which there were 3,815 participants with LVH and 105,549 participants without (one study did not provide the specific number of participants). The duration of follow-up ranged from 3.2 to 11.9 years, with a mean of 7.7 years. The mean age was 55.4 ± 11 years, and 58% of the patients were men. During the follow-up, a total of 3,235 AF events were reported among 115,860 patients, while 258 subjects had LVH. Seven out of the nine manuscripts reported adjusted HR of incidence AF in patients with LVH, and we self-calculated the RR from 2 × 2 tables for the remaining two studies. As illustrated in Figure 2, LVH is related to higher risk of new-onset AF in the population based on mixed blood pressure (RR = 1.65, 95% CI, 1.44–1.89, *I*^2^ = 55.2%) and the population based on hypertension (RR = 1.28, 95% CI, 1.11–1.47, *I*^2^ = 19.9%).

**Table 2A:** Baseline clinical characteristics in patients who were in the studies that investigated the association between LVH and new-onset AF.

**References**	**Study Type**	**Criteria of LVH**	**Groups**	**n**	**Age (years)**
Hamada and Muto (9)	Retrospective cohort study	SV1 + RV5/V6 ≥ 4.5 mV	LVH	1,780	52.4 ± 8.8
			NO LVH	64,204	
Renate et al. (10)	*Post-hoc* analysis of prospective cohort study	-	LVH	182	60.9 ± 9.9
			NO LVH	7,862	
Chamberlain et al. (12)	*Post-hoc* analysis of prospective cohort study	SV3 + RaVL > 2.8 mV (men)or >2.2 mV (women)	LVH	325	-
			NO LVH	14,221	
Jonathan et al. (14)	*Post-hoc* analysis of prospective cohort study	SV1 + RV5/V6 ≥ 3.5 mV	LVH	431	61.4 ± 10.0
			NO LVH	4,511	
Patel et al. (13)	*Post-hoc* analysis of prospective cohort study	Minnesota Code Classification	LVH	224	-
			NO LVH	4,680	
MacFarlane et al. (11)	*Post-hoc* analysis of RCT	Minnesota Code Classification	LVH	117	75.7 ± 3.4
			NO LVH	5,680	
Lehtonen et al. (15)	*Post-hoc* analysis of RCT	SV1 + RV5/V6 ≥ 3.5 mV	LVH	332	58.2 ± 13.8
			NO LVH	2,333	
Verdecchia et al. (17)	Prospective cohort study	SV3 + RaVL > 2.4 mV (men)or >2.0 mV (women)	LVH	424	51 ± 12
			NO LVH	2,058	
Okin (16)	Prospective cohort study	Cornell voltage-duration product ≥2,440 mmms	LVH	8,831	-
			NO LVH		

*LVH, left ventricular hypertrophy; RCT, randomized controlled trial*.

*Cornell voltage-duration product, the product of QRS duration times the Cornell voltage combination (RaVL + SV3, with 6 mm added in women)*.

### The Association Between LVH and the Prognostic of AF

**Figure 3:**
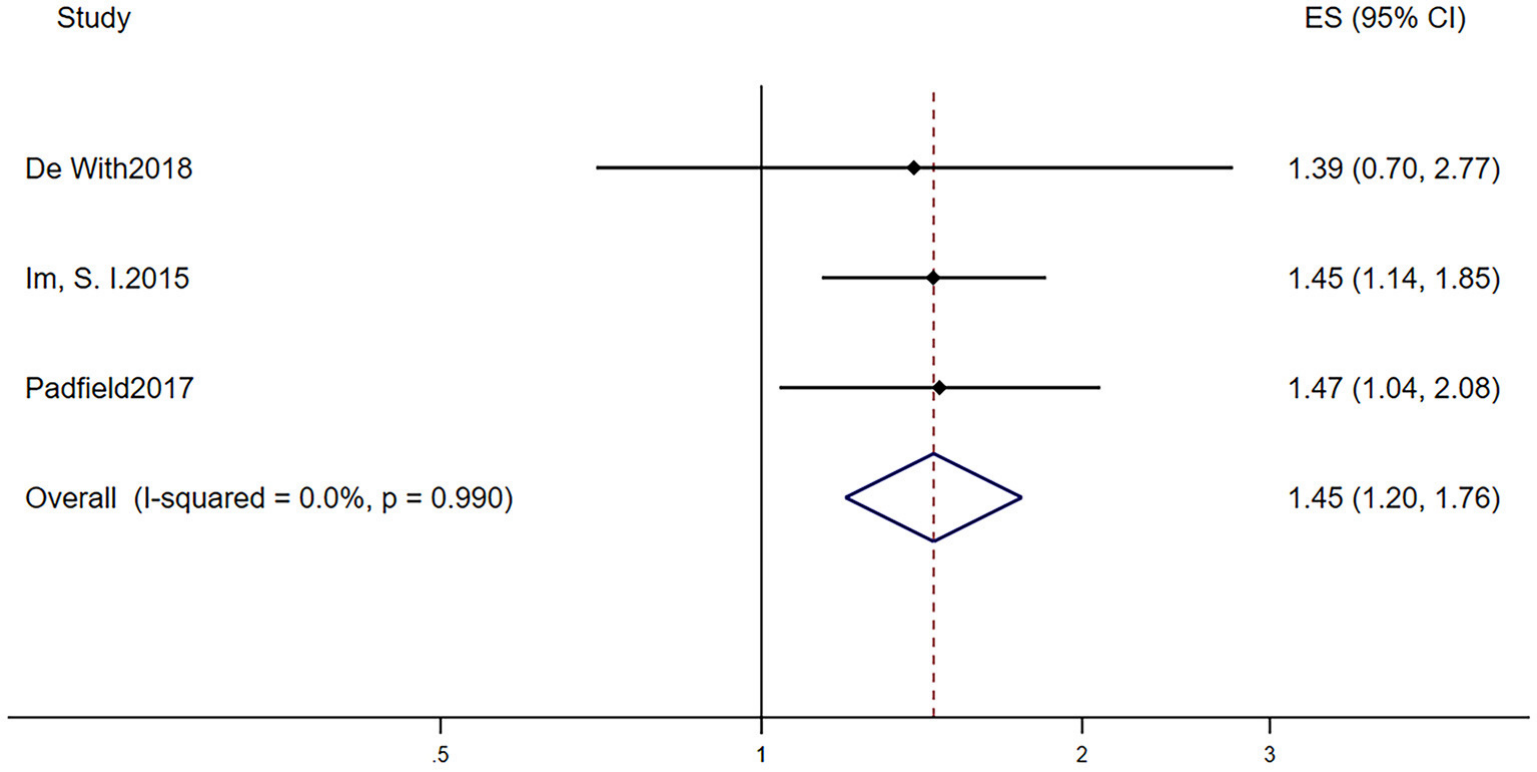
Different risk of AF progression in AF subjects with or without LVH. AF, atrial fibrillation; LVH, left ventricular hypertrophy.

**Figure 4:**
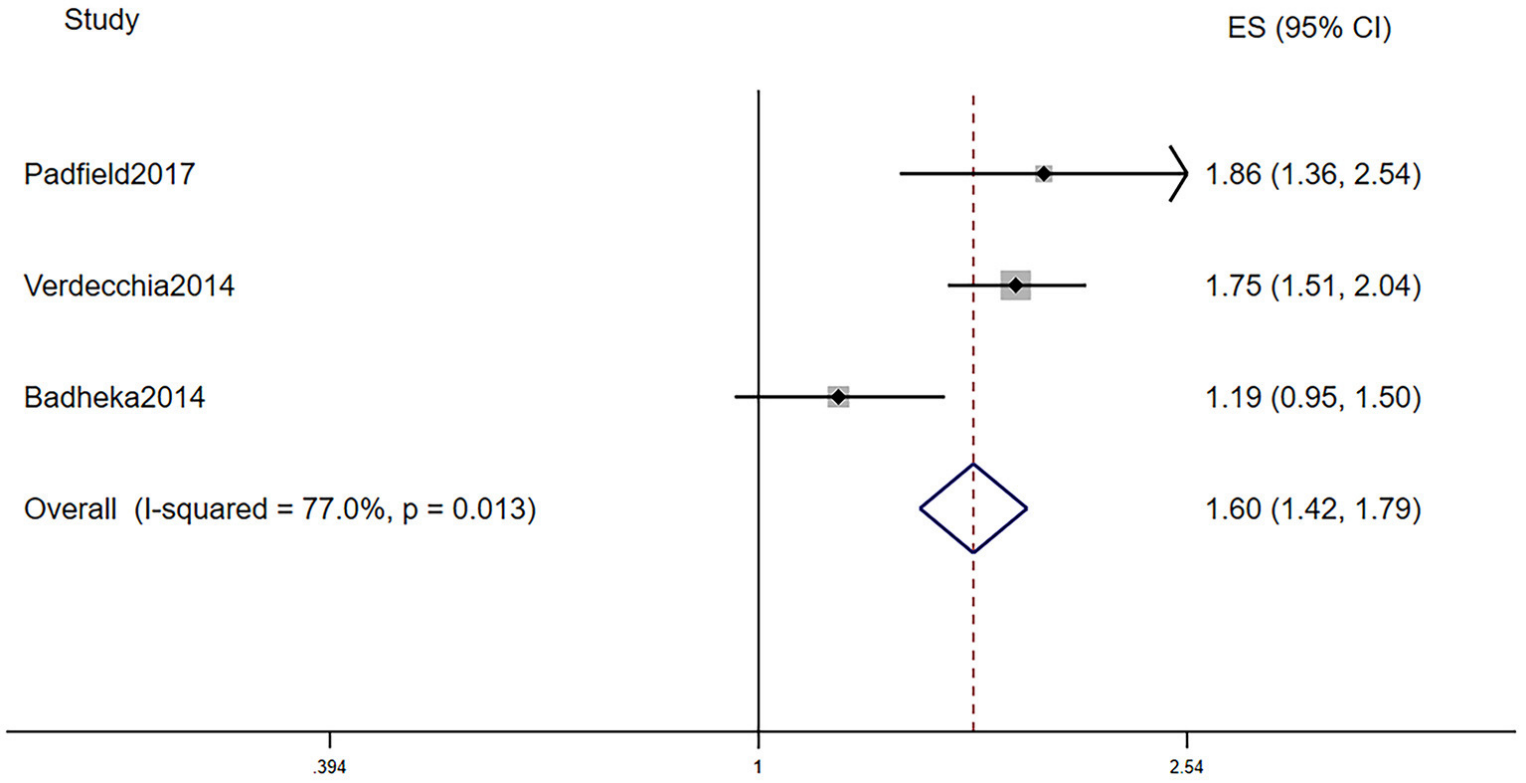
Different all-cause mortality in AF subjects with or without LVH. AF, atrial fibrillation; LVH, left ventricular hypertrophy.

Table 2B displays the baseline characteristics of patients who participated in the five studies that investigated the prognostic role of LVH in AF patients. Due to the limited research assessing the prognostic role of LVH in AF, we included studies that defined LVH via electrocardiogram and echocardiography. Among them, one study defined LVH by electrocardiogram, three studies defined LVH by echocardiography, and one study defined LVH by electrocardiogram or echocardiography. The pooled population consisted of 14,119 subjects, in which there were 3,922 participants with LVH and 10,197 participants without. The duration of follow-up ranged from 2 to 7.2 years, with a mean of 5.0 years. The mean age was 69.4 ± 10.4 years, and 62.8% of the patients were men. In the included five manuscripts, three studies reported the HR of all-cause mortality, and three studies reported the HR of AF progression. Participants with LVH had a higher risk of AF progression (RR = 1.45, 95% CI 1.20–1.76, *I*^2^ = 0%, Figure 3) and all-cause mortality (RR = 1.60, 95% CI 1.42–1.79, *I*^2^ = 77.0%, Figure 4).

**Table 2B:** Baseline clinical characteristics in patients who were in the studies that investigated the association between LVH and the prognosis of AF.

**References**	**Study type**	**Criteria of LVH**	**Groups**	**n**	**Age (years)**	**Male (%)**	**Paroxysmal AF**	**OAC (%)**	**CHA2DS2-VASc Score**	**Follow-Up (years)**
Im et al. (22)	Retrospective cohort study	-	LVH	93	62.8 ± 11.1	256 (60.8)	100%	95(22.6)	1.92 ± 1.4	6.1 ± 4.9
			NO LVH	328						
Verdecchia et al. (5)	*Post-hoc* analysis of RCT	SV3 + RaVL > 2.4 mV (men)or >2.0 mV (women)	LVH	2353	71.3 ± 9	1,413 (60)	15%	1,568 (67)	3.70 ± 1.4	2.0
			NO LVH	8019	71.1 ± 9	5,358 (67)	16%	5,701 (71)	3.36 ± 1.3	
Badheka et al. (20)	*Post-hoc* analysis of RCT	(LVMI) > 95 g/m^2^ in (women)or >115 g/m^2^ (men).	LVH	1373	69.0 ± 8.1	760 (55.4)	49.80%	-	-	3.5 ± 1.3
			NO LVH	732	70.1 ± 8.1	443 (60.6)	57.90%			
Padfield et al. (18)	Prospective cohort study	-	LVH	53	61.2 ± 14.2	289 (38.5)	100%	216 (28.6)	0.22 ± 0.41	6.35
			NO LVH	700						
De With et al. (21)	Prospective cohort study	(LVMI) > 95 g/m^2^ in (women)or>115 g/m^2^ (men).	LVH	50	49 ± 9	354 (76)	70%	-	1 ± 0.5	7.2
			NO LVH	418						

*LVH, left ventricular hypertrophy; RCT, randomized controlled trial; LVMI, left ventricular mass index*.

**Figure 5:**
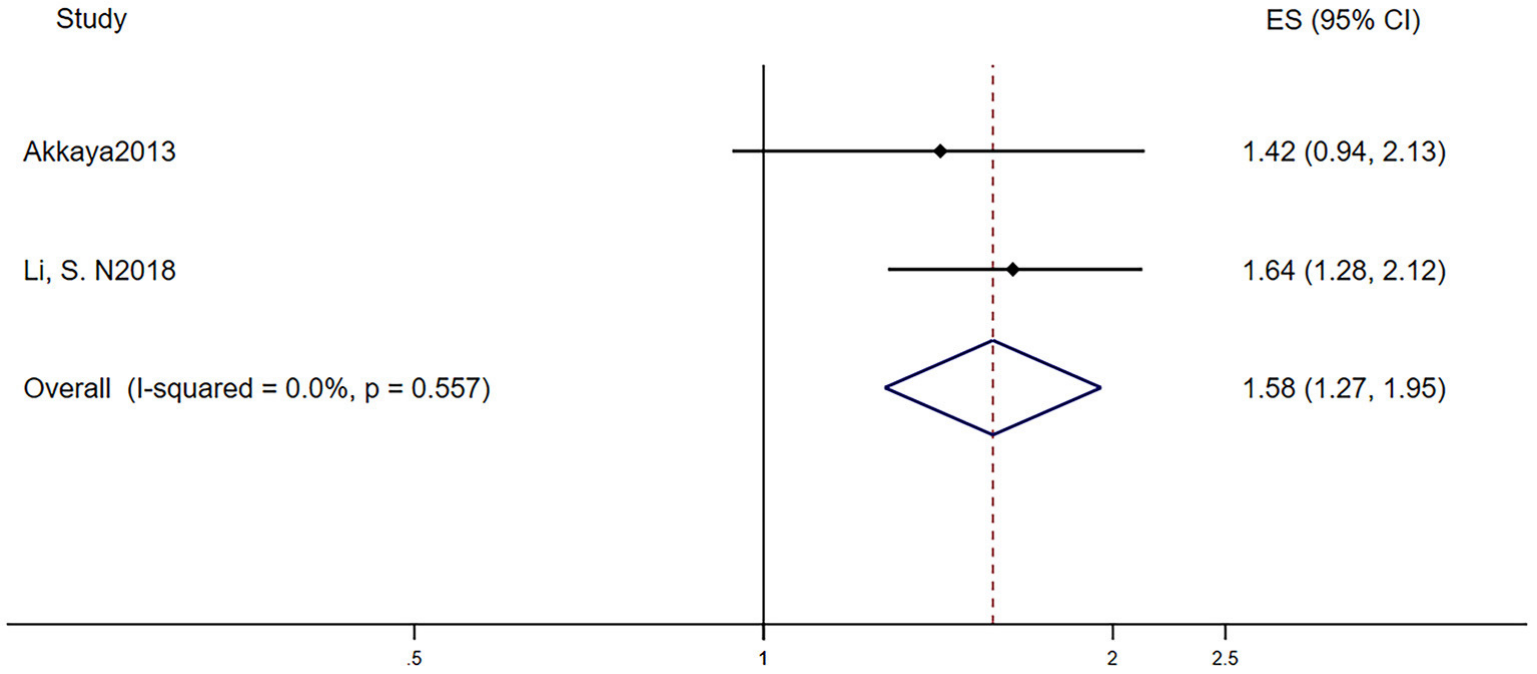
Different risk of recurrence after catheter ablation in AF subjects with or without LVH. AF, atrial fibrillation; LVH, left ventricular hypertrophy.

Table 2C reports the characteristics of patients in the two studies that investigated the different recurrence risk of AF after catheter ablation with or without LVH. One study defined LVH by electrocardiogram, and the other study defined LVH by echocardiography. The pooled population consisted of 777 subjects, in which there were 318 participants with LVH and 459 participants without. The mean follow-up was 26.5 months. The mean age was 62.8 ± 11.3 years, and 70.1% of the patients were men. During the mean follow-up of 26.5 months, 131 patients developed AF recurrence in 459 participants without LVH, while 147 patients developed AF recurrence in 318 participants with LVH. In other words, AF patients with LVH have a higher risk of developing AF recurrence after the surgery than those without LVH (RR = 1.58, 95% CI 1.27–1.95, *I*^2^ = 0%, Figure 5).

**Table 2C:** Baseline clinical characteristics in patients who were in the studies that investigated the association between LVH and the recurrence of AF after catheter ablation.

**References**	**Study type**	**Criteria of LVH**	**Groups**	**n**	**Age (years)**	**Male (%)**	**Paroxysmal AF (%)**	**Follow-Up**
Akkaya et al. (23)	Retrospective cohort study	LVMI > 104 g/m^2^ (women)Or >116 g/m^2^ (men)	LVH	111	65.4 ± 11.3	95 (77.9)	49.90%	11.1 ± 3.8 months
			NO LVH	246	64.8 ± 12.3	165 (58.5)		
Li et al. (24)	Prospective cohort study	Romhilt-Estes point score≥5 points	LVH	207	61.6 ± 10.8	144 (66.1)	100%	42 months
			NO LVH	213	60.2 ± 9.8	141 (64.7)		

*LVH, left ventricular hypertrophy; LVMI, left ventricular mass index*.

## Discussion

One of our findings was that LVH assessed by ECG was a significant predictor of AF. AF has a high prevalence worldwide and was related to many complications and higher all-cause mortality rates (1). ECG is a relatively inexpensive, convenient, and widely available tool for screening disease. Hence, we sought to find out whether ECG could predict the development of AF independently. As illustrated in Figure 2, subjects with ECG-LVH in both subgroups stratified by the baseline blood pressure were at a higher risk for the incidence of AF. Hypertension not only is a risk factor of cardiovascular complications but also increases the risk for the development of atrial fibrillation. Okin et al. (25) determined that the regression of ECG-LVH would decrease the incidence of new-onset AF in patients with hypertension. These findings suggest that therapy aimed to decrease the LVH may be required. However, this result was heterogeneous. Other factors may account for the finding. First, although cases of LVH were all assessed by ECG, there were varying criteria. Jonathan et al. (14) analyzed 11 of ECG-LVH criteria in terms of their associations with incident AF and found that only 3 of the 11 ECG-LVH criteria had significant associations with AF after adjustment. Second, in the multivariate analysis, the models were different; some studies were adjusted only for sex and age, while some were adjusted for all risk factors, and difference in baseline characteristics and sample sizes would both contribute to the heterogeneity. Finally, some studies were *post-hoc* analyses based on other trials, and this is a potential source of uncontrolled bias and confounding factors.

Another remarkable finding was that AF in the context of LVH was associated with worse outcomes. These patients were more susceptible to AF progression (i.e., from paroxysmal AF to persistent or permanent AF) and death. AF is considered as a progressive disease; the overall progression rate is >5% per annum (26). Benjamin et al. (27) indicated that persistent AF would result in a higher risk of thromboembolic events and worse survival compared with those with paroxysmal AF. To identify patients who were likely to progress to sustained AF at 1-year follow-up, de Vos et al. (28) developed the HATCH score (the acronym stands for hypertension, age [75 years and older), transient ischemic attack or stroke (2 points), chronic obstructive pulmonary disease, and heart failure (2 points)]. They found that nearly half of subjects with a HATCH score higher than 5 and only 6% of patients with a HATCH score of 0 progressed to sustained AF within 1 year. To evaluate the accuracy of the HATCH score, Barrett et al. (29) conducted a retrospective cohort including 253 AF patients and found that 61 participants progressed to sustained AF within 1 year. Nevertheless, only two participants had scores >5, suggesting that the HATCH score did not identify patients who were actually at high risk for AF progression. Refinement of this decision aid is needed. It is feasible to add LVH as a predictor of AF prognosis, and that would be definitely helpful for physicians to make clinical decision that may improve the progression of AF.

AF is independently associated with a 2-fold increased risk of all-cause mortality in women and a 1.5-fold increase in men (30). We demonstrated that patients with AF in the context of LVH had a 1.46-fold increased risk than those without LVH. Furthermore, Badheka et al. (20) divided participants with LVH into three groups (mildly abnormal, moderately abnormal, or severely abnormal) according to the American Society of Echocardiography criteria. They found that higher LV mass correlated with greater degrees of association between LVH and all-cause mortality. In addition, Verdecchia et al. (5) found that excess all-cause mortality associated with LVH was greater in patients with lower (from 0 to 2) than higher (>3) CHA_2_DS_2_-VASc scores, suggesting that ECG-LVH may be more suitable as a prognostic marker in patients with lower CHA_2_DS_2_-VASc scores. To sum up, LVH is a prognostic predictor of all-cause mortality in those with severe LVH and low CHA_2_DS_2_-VASc scores. Consequently, it is necessary and favorable to screen out the high-risk groups, and a corresponding therapy is needed. There are two strategies for treatment of atrial fibrillation: one is rhythm control with antiarrhythmic drugs to maintain sinus rhythm, and the other is rate control, allowing atrial fibrillation to persist. In patients with AF in the context of LVH, Badheka et al. (20) reported that LVH was a significant independent predictor of mortality, especially in patients managed with rhythm control, suggesting that strict rate control may be associated with better outcomes than is rhythm control. Nevertheless, there is significant heterogeneity in our result. When we pooled the results using random-effects models or excluded any of the studies, the heterogeneity remained. The incorporation of studies using either echocardiographic or electrocardiographic assessment of LVH may account for this finding. Moreover, although the result was identified using multivariable analysis, the adjusted Cox model was not totally the same in the four trials; differences of patterns of AF, length of follow-up, CHA_2_DS_2_-VASc scores, and other baseline characteristics all generate heterogeneity. Finally, the design structure of the trials and *post-hoc* analysis may contribute to the heterogeneity.

Catheter ablation of AF, generally a second-line treatment, is more effective than antiarrhythmic drug therapy in rhythm control (31). However, the recurrence of AF within 1 year was not uncommon. Previous studies (32) demonstrated that left atrial fibrosis predicted recurrence. AF patients with LVH also had a higher degree of left atrial fibrosis (33). Compared with screening for left atrial fibrosis, screening for LVH is much more convenient and has low cost. More large randomized controlled trials are needed to demonstrate the independence of LVH as a predictor in the recurrence of AF.

## Limitation

The present study has some limitations, including differences among the study designs, sample characteristics, and quality of the included studies. The difference criteria of LVH may restrict the credibility of the results; more large randomized controlled studies are highly needed.

## Data Availability Statement

The original contributions presented in the study are included in the article/supplementary material, further inquiries can be directed to the corresponding authors.

## Author Contributions

KJ and HZ make the conception and design of the review. HX make the analysis and interpretation of the data as well as drafting the manuscript. YY, YX, ZC, YP, and JW make the critical revision of the manuscript for important intellectual content. All authors contributed to the article and approved the submitted version.

## Conflict of Interest

The authors declare that the research was conducted in the absence of any commercial or financial relationships that could be construed as a potential conflict of interest.

## Publisher's Note

All claims expressed in this article are solely those of the authors and do not necessarily represent those of their affiliated organizations, or those of the publisher, the editors and the reviewers. Any product that may be evaluated in this article, or claim that may be made by its manufacturer, is not guaranteed or endorsed by the publisher.
